# Prenatal Brain Damage in Preeclamptic Animal Model Induced by Gestational Nitric Oxide Synthase Inhibition

**DOI:** 10.1155/2011/809569

**Published:** 2010-12-27

**Authors:** Begoña Pellicer, Sonia Herraiz, Antonio Leal, Carlos Simón, Antonio Pellicer

**Affiliations:** ^1^Servicio Ginecologia y Obstetricia, Hospital de Manises, C/Roses s/n, 46940 Valencia, Spain; ^2^Valencian Node of the National Stem Cell Bank, Prince Felipe Research Centre, 46012 Valencia, Spain; ^3^Fundación IVI-Instituto Universitario IVI, Universitat de València, 46015 València, Spain

## Abstract

Cerebral palsy is a major neonatal handicap with unknown aetiology. There is evidence that prenatal brain injury is the leading cause of CP. Severe placental pathology accounts for a high percentage of cases. Several factors predispose to prenatal brain damage but when and how they act is unclear. The aim of this paper was to determine if hypoxia during pregnancy leads to damage in fetal brain and to evaluate the localization of this injury. An animal model of chronic hypoxia produced by chronic administration of a nitric oxide synthase inhibitor (L-NAME) was used to evaluate apoptotic activity in fetal brains and to localize the most sensitive areas. L-NAME reproduces a preeclamptic-like condition with increased blood pressure, proteinuria, growth restriction and intrauterine mortality. Apoptotic activity was increased in L-NAME brains and the most sensitive areas were the subventricular and pallidum zone. These results may explain the clinical features of CP. Further studies are needed.

## 1. Introduction

Two of every 1000 children born alive will develop some degree of cerebral palsy (CP). Even though its aetiology is unknown, there is increasing evidence that prenatal brain injury is the leading cause of CP rather than peripartum brain insult [1], especially prenatal hypoxic-ischemic encephalopathys [2]. Prematurity, hypoxia, ischemia, and inflammation are clinical factors that predispose to prenatal brain damage, but when and how they act is unclear. 

Preeclampsia (PE) affects 8–10% of pregnancies worldwide [3]. It has been shown in several studies that children born from preeclamptic mothers may have a various degree of neurodevelopmental delay. Long-term effects have been reported in newborns with growth restriction added to a preeclamptic [4, 5]. Severe placental pathology, as preeclampsia, accounts for 21 percent of all cases of cerebral palsy in an Australian cohort [6]. The mechanism of brain damage caused by maternal hypoxia in offspring is not clear. It has been reported that acute prenatal hypoxia altered the structural and functional properties of cell membranes and initiated apoptotic gene transcription [7], but most previous studies have employed models of acute cerebral hypoxia-ischemia in postnatal animals [8].

The location of the neurological damage should explain the clinical features in cerebral palsy, but neonatal imaging findings in hypoxic-ischemic injury are highly variable and depend on a number of factors, including brain maturity, severity and duration of insult, and type and timing of imaging studies [9]. De Vries et al. described the existence of periventricular hemorrhages in fetuses with chronic hypoxia and PE [10, 11]. However, no clear correspondence has been described between the location of the haemorrhage and the long term outcome and handicaps the neonate will develop.

None of the areas most sensitive to hypoxic insult have yet been clarified. Severe asphyxia in animals at term showed that progenitors within the subventricular zone (SVZ) are vulnerable to this insult [12], and also a neuronal loss in the hippocampus after prenatal hypoxia has been described [13, 14]. 

The main aim of our study was to determine if hypoxic situation during pregnancy leads to a damage in the fetal brain, and to evaluate the localization of this prenatal injury. We used an animal model for this purpose, in which we reproduced a PE-like condition by chronic inhibition of nitric oxide synthase (NOS) during gestation in rats. It results in pathological changes similar to those observed in women with preeclampsia, such as severe renal vasoconstriction, proteinuria, thrombocytopenia, and intrauterine growth restriction [15, 16].

## 2. Material and Methods

### 2.1. Animals and Study Design

Female Wistar rats approximately 10 weeks of age and weighing 200–250 g, were maintained at constant room temperature (22°C), 60% relative humidity, for a 12 hour dark-light cycles with standard feeding. Animals were randomly allocated in two experimental groups. 

A first group were animals had food and tap water ad libitum was the control group (*n* = 15), and a second group were animals receives 50 mg/Kg/day of L-NAME, a nitric oxide synthase inhibitor, dissolved in drinking water since day 0 of pregnancy (E0) were named L-NAME group (*n* = 15).

To study the effect of NOS inhibition on the foetuses, we established sacrifice days which corresponded with the first, second, and third trimesters in human pregnancies [17, 18]. Five mothers by group were sacrificed on gestational days E8, E11, and E18 and the foetal cerebral tissues were fixed for further analysis. 

The study was performed in accordance with European Directive 86/609/CEE and the guidelines of the National Institutes of Health (NIH) for the care and use of laboratory animals. The study protocol used was approved by the ethics committee of the Centro de Investigación Príncipe Felipe (CIPF), Valencia, Spain.

### 2.2. Systolic Blood Pressure (SBP)

We obtained SBP values using a noninvasive automatic blood pressure analyzer (NIPREM 564, Cibertec, Madrid, Spain) attached to the rat's tail after previously dilating the vessels with a stove (36-37°C) for 5 minutes, as described elsewhere [19, 20]. The mean of 5 readings from each animal was considered as the individual SBP value. The measurements were performed on gestational days E1, E8, E11 and E18 in both groups. 

### 2.3. Perinatal Outcome

Various approaches were followed to analyse the weight of the different structures. On gestational days E8 and E11, we measured the weight of a section of the pregnant uterus between the implantation sites, which included both the foetuses and the associated uterine walls [20]. On gestational day E18 the crown-rump (CRL) and occipital-snout lengths (OSL) were measured. The placental weight was recorded, and foetal organs were macroscopically reviewed in 5 foetuses per group to rule out congenital anomalies. Cerebral and hepatic organs were extracted and weighted. 

Antenatal and perinatal mortality were also studied. Intrauterine mortality was calculated on gestational day E8, E11 counting resorptions number and on E18 by evaluating the number of atrophic fetuses in the uterine horns of each mother. 

### 2.4. Immunostaining of Brain Samples from E18

At embryonic day 18 fetuses were decapitated, and the brain was dissected out. Six fetuses per group randomly selected from all the obtained fetuses, were used. After PFA 4% fixation of cerebral tissue, it was maintained in a 30% glucose solution during 24 hours to cryoprotect the tissue prior to freezing. Serial coronal slices of tissue were obtained every 20–25 micrometers.

An immunohistochemistry analysis was then performed to detect cleaved caspase-3 activity and tubuline beta-III in fetal brains.

Sections were incubated for 45 minutes with blocking solution containing 5% normal goat serum (NGS, Sigma St. Louis, MO, USA) and 5% non-fat dry milk, then were incubated overnight at 4°C with anticaspase-3-activated antibody (1 : 200, cell signalling technology).

An antirabbit biotinylated secondary antibody (1 : 400) was used. Afterwards the Vectastain Elite ABC kit the samples were used and after further washes in PBS, cleaved caspase-3 labelled cells were visualized using diaminobenzidine (DAB) plus nickel sulphate solution (Vectastain, Vector laboratories) yielding a black reaction product. After visualization of Caspase-3 immunoreactivity, sections were rinsed in PBS and incubated with one drop of Avidin-D blocking solution for 15 minutes (Avidin-Biotin blocking solution kit, Vector Laboratories) and rinsed with PBS. Then sections were incubated overnight at 4° with primary antibody anti- tubulin-beta-III (1 : 250). 

Following washes with PBS, sections were blocked with 3% milk in the wash buffer and incubated 1 hour with a secondary antimice antibody (1 : 250). After wards ABC system was, used and tubuline-beta-III labelled cells were visualized using DAB solution. 

Sections were dehydrated and mounted.

Quantitative analysis of apoptotic positive cells was done on a Leica microscope equipped with a video camera (Leica DMR 6000, Leica Microsystems CMS GMBH, Wetzlar, Germany). Inside positive areas, microphotographs at 20x were analysed in order to count and compare apoptotic patterns. 

The areas of the CNS that were analysed were: subventricular zone (B11-B12), pallidum zone (B1-B2), preoptic area-anterior hypothalamus (H24-H25), hypothalamic neuroepithelium (H1), amygdala (A7-A8-A9-A10), and striatum (B3–B13-B14–B23). Cell counting was performed using the Image J, an image analyzer software. For each animal, 4 images by area were analyzed to determine the number of apoptotic cell in these zones.

### 2.5. Data Analysis

The results are shown as the mean ± standard error of the mean (SEM). Comparisons between multiple groups were performed using ANOVA and a Newman-Keuls post hoc test. An unpaired *t*-test was done to make comparisons between the two groups. Significant differences were considered to be *P* < .05. The GraphPad Prism 4.0. (GraphPad Software INC. La Jolla, CA, USA) and the SPSS 17.0 (SPSS Inc. Chicago, IL, USA) softwares were used.

## 3. Results

**Figure 1:**
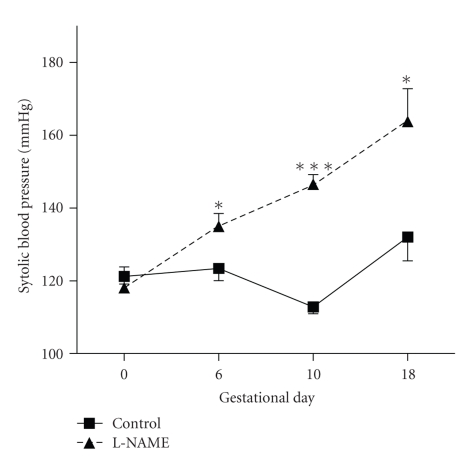
Systolic blood pressure evolution in pregnant rat. Control group shows levels that remain constant along pregnancy except day 10, when SBP falls, like in human pregnancies. L-NAME group showed an increase in SBP values in pregnancy, which was significant from day 6. (E6 *P* < .05; E10 *P* < .0001; E18 *P* < .05).

SBP showed a significant increase in the L-NAME group in all the evaluated moments throughout the pregnancy when compared with the control group, as shown in Figure 1, and this significance was clear since day 6 of pregnancy (E6 *P* < .05, E11 *P* < .0001 and E18 *P* < .05) Also, it showed the presence of proteinuria. 

**Figure 2:**
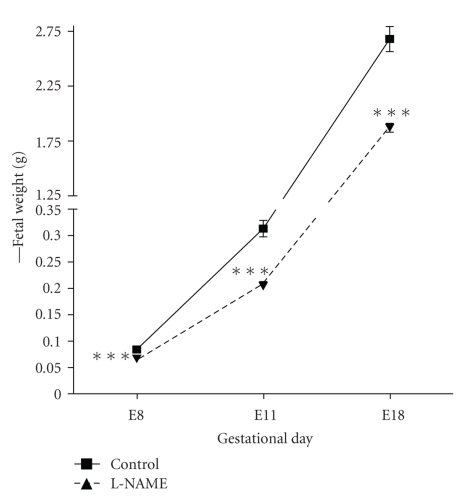
Fetal weight throughout pregnancy. L-NAME group shows a decrease in all evaluated moments. E8, E11, and E18 *P* < .0001.

When we analyzed weight gain and biometrical measurements we found a decrease in fetal weight in L-NAME group on E6 (0,084 ± 0,003 g versus 0,066 ± 0,003 g), E11 (0,313 ± 0,015 g versus 0,204 ± 0,008 g), and E18 (2,67 ± 0,11 g versus 1,87 ± 0,04 g) (*P* < .0001, Figure 2). Also, a placental weight decrease over a 20% compared to control was observed (0,67 ± 0,03 g in control and 0,54 ± 0,02 g in L-NAME group, *P* < .001). Macroscopic analysis of fetal organs found that, like the placenta, there was a decrease in fetal liver in L-NAME group (*P* < .05).

**Figure 3:**
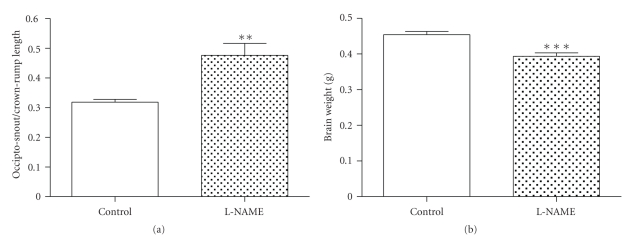
A- OSL/CRL index, a significant increase was observed in L-NAME group (*P* < .001). B- Fetal Brain weight in E18, a decrease was detected in L-NAME group (*P* < .0001).

There was no significant difference in CRL and OSL between groups. However, when we calculated the OSL/CRL index, there was a statistically significant increase in the L-NAME group, which represents smaller heads in this group (OSL/CRL was 0,318 ± 0,009 and 0,476 ± 0,040 in control and L-NAME group, resp., *P* < .001) (Figure 3(a)). 

**Figure 4:**
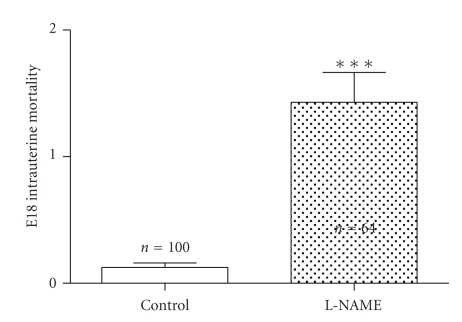
Comparison of intrauterine mortality present in the groups of study. A clear increase was observed in L-NAME group (*P* < .0001).

Regarding the measurements, there was a significant difference in brain weight between control and L-NAME treated fetuses (0,454 ± 0,008 g and 0,393 ± 0,009, resp., *P* < .0001; Figure 3(b)), the brain/fetal weight showed no significant differences, althought there was a trend to an increase index in the L-NAME group. Finally, in L-NAME group there was a 7-fold higher intrauterine mortality than in control group. (Figure 4, *P* < .0001).

### 3.1. Analysis of Apoptosis in Fetal Brain

**Figure 5:**
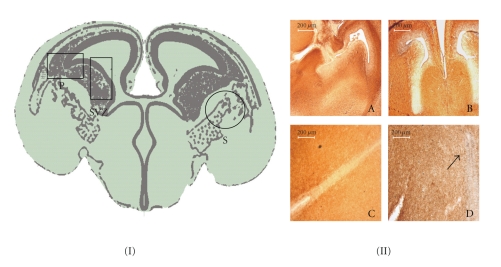
(I) E18 rat brain coronal scheme, black box indicates an increase in apoptotic activity, and black circle shows a decrease (*P* < .05) in L-NAME compared to control group. (II) A-B, Overview at lower magnification of E18 CNS in control (A) and L-NAME group (B) ×5. Panels C-D shown at high magnification: positive cleaved caspase 3 immunostained (black arrows) in neuroepithelial cells of anterior hypothalamus in PE group (D) ×20.

**Figure 6:**
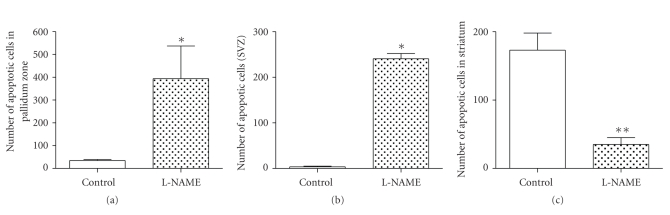
Analysis of apoptotic activity in fetal brain. A-pallidum zone (*P* = .0028). B-subventricular zone (*P* = .0024). C-striatum (*P* = .03).

L-NAME group showed an increased pattern of apoptotic activity of the fetal brain versus the control group (*P* < .05, Figure 5). When we analysed specific cerebral areas, we found that in the pallidum zone of the fetal brain there was a significant increase in the apoptotic activity detected as compared to the control group (*P* = .0028) (Figure 6(a)). In the subventricular zone there was also a significant increase in the apoptotic activity (*P* = .0024) (Figure 6(b)). 

However, in the striatum area and in the amygdala 7 there was a decrease in the number of apoptotic cells, being significant only in the striatum area (*P* = .036, Figure 6(c)). Finally, there was a nonsignificant decrease in the apoptotic activity in the preoptic area, which corresponds to the anterior hypothalamus.

## 4. Discussion

The present study has examined how chronic hypoxia model since the beginning of the pregnancy via NOS inhibition may produce growth restriction in fetuses, with an increased mortality, and it also produces apoptotic damage in some cerebral areas analysed in the third trimester of pregnancy. The major aim of our study was to determine the presence of apoptotic damage in fetal brain specimens. Even though this prenatal insult may represent some clinical features in the newborn or adult, we concentrated our efforts in the histopathological analysis of the damage.

 In this model, we observed an increase in SBP plus the presence of proteinuria in addition to the difference in biometrical measurements. These results are consistent with previous reports that demonstrated that NOS inhibition results in a PE-like syndrome and a decrease for gestational age fetuses in rats [16], probably due to blood flow reduction through both uterine arteries during pregnancy [21]. However, the mechanisms by which chronic hypoxia acts to reduce this blood flow remain unknown [22]. 

It is interesting that fetal weight is reduced, while fetal length is not affected. In this sense, there also been studies that human fetuses under chronic hypoxia conditions suffer from asymmetrical growth restriction, especially in the abdominal circumference (AC) measurements when compared with the rest of biometrical parameters [23]. Also, AC measurements measured midgestation are the best predictors for birth weight [24]. A small placenta and increased mortality, as seen in our model, are also common findings in this kind of fetuses. As described above, it could be a good model to determine certain aspects of the developing fetus under similar conditions than pregnancies complicated with preeclampsia.

Our main objective was to analyse the possibility of intrauterine fetal brain damage under conditions described above independently of labour injury. 

A significant brain damage after exposure to chronic hypoxia was found. Immature brain exposure to chronic NOS inhibition should be carefully considered, as L-NAME is not a selective NOS endothelial inhibitor. However, it has been seen that the NO is harmful for the developing brain, and we must assume that the total amount of this substance is decreased in our model.

 We have localized significant damage in the SVZ and in the pallidum zone (PZ). We evaluated the loss of neuronal cells via apoptosis, as in recent years an increasing body of evidence has showed that after cerebral hypoxia-ischemia neuronal cell damage occurs not only via necrosis, but also through apoptotic processes [25]. The main mechanism of damage is still unknown. In this sense, there seems to be a special vulnerability of a specific subpopulation of cells, the oligodendrocytes, that have been located in the SVZ after acute perinatal brain injury [12]. It has been also recently described that the brain damage caused by chronic hypoxia does not depend directly on oxygen deprivation, but that it is also an adaptative response via a cytokine function that becomes maladaptive. However, in our study we did not analyse the presence of cytokines in fetal brains, as it was not our major aim [26].

Regarding the PZ, this area is the precursor of planning and inhibition of voluntary movements, and it has not been related with perinatal hypoxic damage. Rees et al. [27] found cortical disturbances in a sheep model of chronic hypoxia since midgestation. These disturbances were found in a more developed brain. We studied immature brains under a nonphisyological hypoxic mechanism that must develop postnatally, and this should be taken in account in further studies.

## 5. Conclusions

In summary, our study shows that chronic hypoxia during pregnancy produces an apoptotic damage in the cerebral tissues. These results have showed us that the most sensitive areas to chronic hypoxia prior to birth were SVZ and PZ when compared to the control group. The consequences of this damage and the postnatal expression of this damaged areas need further specific studies.
